# Clinical spectrum transition and prediction model of nonalcoholic fatty liver disease in children with obesity

**DOI:** 10.3389/fendo.2022.986841

**Published:** 2022-08-31

**Authors:** Xuelian Zhou, Xiufu Lin, Jingnan Chen, Jiaqi Pu, Wei Wu, Zhaoyuan Wu, Hu Lin, Ke Huang, Li Zhang, Yangli Dai, Yan Ni, Guanping Dong, Junfen Fu

**Affiliations:** The Children’s Hospital, Zhejiang University School of Medicine, National Clinical Research Center for Child Health, Hangzhou, China

**Keywords:** children, nonalcoholic fatty liver disease, clinical spectrum, prediction model, obesity

## Abstract

**Objective:**

This study aims to outline the clinical characteristics of pediatric NAFLD, as well as establish and validate a prediction model for the disease.

**Materials and methods:**

The retrospective study enrolled 3216 children with obesity from January 2003 to May 2021. They were divided into obese without NAFLD, nonalcoholic fatty liver (NAFL), and nonalcoholic steatohepatitis (NASH) groups. Clinical data were retrieved, and gender and chronologic characteristics were compared between groups. Data from the training set (3036) were assessed using univariate analyses and stepwise multivariate logistic regression, by which a nomogram was developed to estimate the probability of NAFLD. Another 180 cases received additional liver hydrogen proton magnetic resonance spectroscopy (1H-MRS) as a validation set.

**Results:**

The prevalence of NAFLD was higher in males than in females and has increased over the last 19 years. In total, 1915 cases were NAFLD, and the peak onset age was 10-12 years old. Hyperuricemia ranked first in childhood NAFLD comorbidities, followed by dyslipidemia, hypertension, metabolic syndrome (MetS), and dysglycemia. The AUROC of the eight-parameter nomogram, including waist-to-height ratio (WHtR), hip circumference (HC), triglyceride glucose-waist circumference (TyG-WC), alanine aminotransferase (ALT), high-density lipoprotein cholesterol (HDL-C), apolipoprotein A1(ApoA1), insulin sensitivity index [ISI (composite)], and gender, for predicting NAFLD was 0.913 (sensitivity 80.70%, specificity 90.10%). Calibration curves demonstrated a great calibration ability of the model.

**Conclusion and relevance:**

NAFLD is the most common complication in children with obesity. The nomogram based on anthropometric and laboratory indicators performed well in predicting NAFLD. This can be used as a quick screening tool to assess pediatric NAFLD in children with obesity.

## Introduction

Nonalcoholic fatty liver disease (NAFLD) is histologically defined as fatty infiltration ≥ 5% hepatocytes in the absence of other known causes of fatty liver, with a spectrum of diseases including nonalcoholic fatty liver (NAFL), nonalcoholic steatohepatitis (NASH), liver fibrosis, and cirrhosis (1). NAFLD has increased in prevalence in concert with the global pandemic of obesity and type 2 diabetes mellitus (T2DM), which has become the most common form of chronic liver disease all over the world (2, 3). The prevalence of pediatric NAFLD in America is about 3% to 11% (4), while the prevalence in Asia and China is about 6.3% and 3.4% respectively (5). The early onset of obesity significantly increases the risk of developing NAFLD in adolescence (6). The prevalence of NAFLD in children and adolescents has increased by 62% over the past ten years, nearly one-third of boys and one-fourth of girls suffered from NAFLD in obese children (7, 8). The increased incidence of pediatric NAFLD is closely associated with an unhealthy diet, such as industrialized products, fried foods, and sweetened beverages (9). It is suggested that the early onset of NAFLD may lead to advanced liver disease earlier in adulthood, and the impacts of the bad habits developed during childhood are difficult to overcome in the future. The early onset of NAFLD and insulin resistance seems to be responsible for increased risk of cardiovascular events and promote the progression of T2DM and chronic kidney disease (10–14).

Most pediatric cases are NAFL, a relatively benign, non-progressive clinical course, while 17-38% of NASH will progress to fibrosis and cirrhosis (15, 16). It is estimated that the overall and liver-specific mortality in NAFLD populations are 11.77 and 0.77 per 1000 person-years, whereas they are 25.56 and 15.44 per 1000 person-years among NASH populations (3). Therefore, comprehensive screening of NAFL (potential risk group) and early identification of NASH (high-risk group) in childhood are key issues in preventing and treating the chronic progression of liver disease. Efficient and cost-effective assessment approaches for risk stratification of pediatric NAFLD for these 2 critical end points are urgently needed.

Liver biopsy is the gold standard for the diagnosis of NAFLD but the invasiveness, cost, and sampling variability limit its application in clinics. Hydrogen proton magnetic resonance spectroscopy (^1^H-MRS) can quantitatively measure liver fat content in a highly accurate and precise manner (17). Nevertheless, MRS is time-consuming and costly. Ultrasonography is still the most commonly used imaging technique for NAFLD screening in pediatrics due to its non-invasive and economical nature. However, the sensitivity of ultrasound for the detection of mild steatosis is 60.9%-65% (18, 19). Thus, alternative noninvasive strategies, such as serum biomarkers (ALT/AST ratio, APR Index, BARD score, NAFLD fibrosis score, FIB-4 index, exosomal miRNAs), and imaging techniques (elastography) have made great strides over the past decade (20, 21). Other cardiometabolic risk factors, such as triglyceride glucose-waist circumference(TyG-WC), triglyceride glucose-body mass index(TyG-BMI), triglyceride glucose-waist-to-height ratio(TyG-WHtR), waist-to-hip ratio(WHR), waist-to-height ratio(WHtR), and homeostatic model assessment of insulin resistance(HOMA-IR) have also been reported to be suitable predictors of NAFLD (22–24). However, the sensitivity and specificity of a single index still need to be improved.

Non-invasive, fast, and accurate prediction models based on biochemical and anthropometry parameters were lacking in pediatric NAFLD. Nomogram is a graphical presentation of a clinical prediction model, which is helpful in the early identification of high-incidence diseases and can be widely used in clinics, and even in primary hospitals. This study aims to explore the clinical spectrum and establish a nomogram for predicting childhood NAFLD.

## Methods

### Subjects

**Figure 1:**
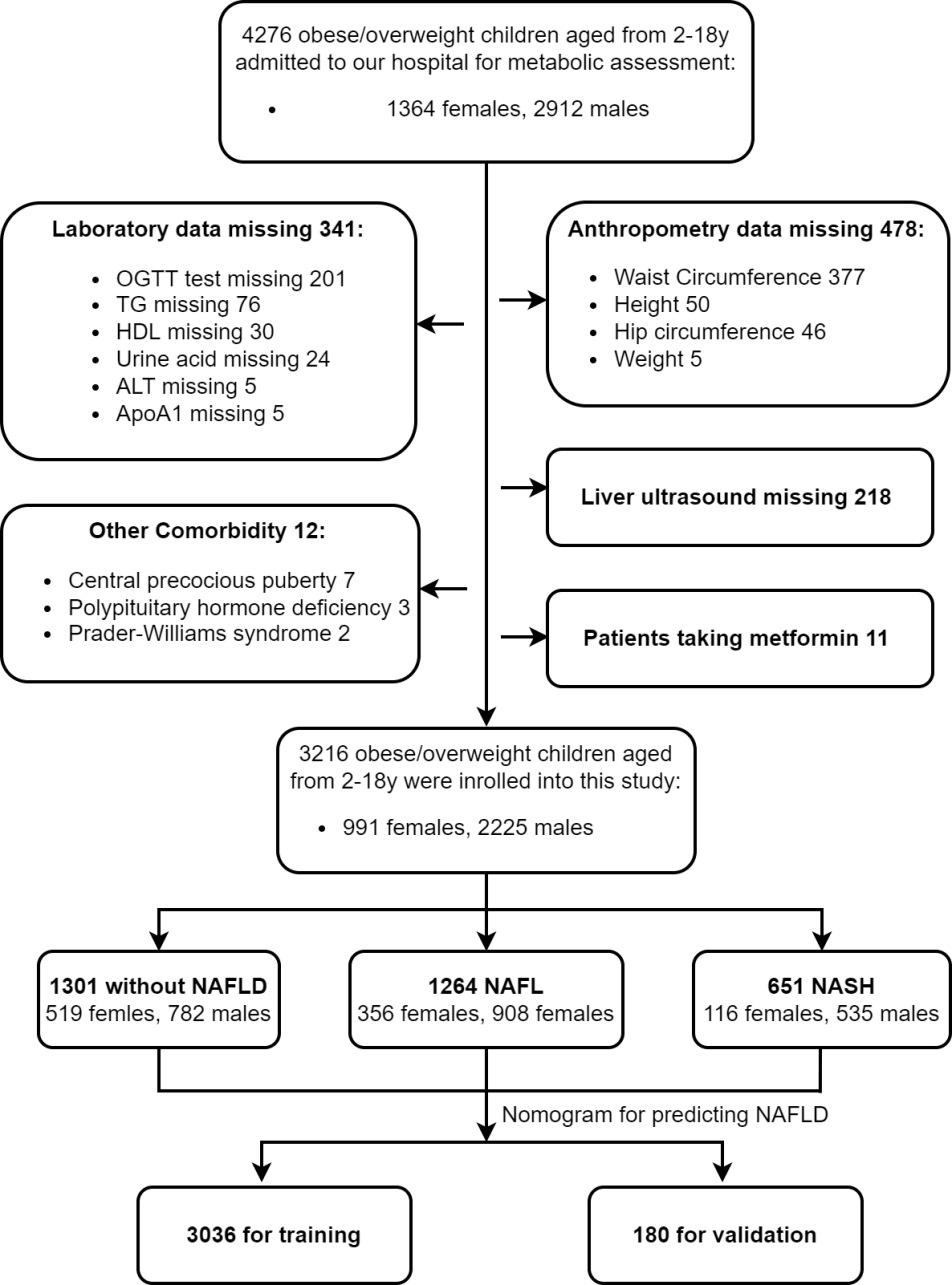
Flow chart. We identified a total of 4276 children with obesity from Jan 2003 to May 2021 who came to our hospital for metabolic assessment. 3216 children were enrolled in this study according to the above exclusion criteria. They were divided into three groups according to liver ultrasound and serum ALT concentration, and their clinical characteristics were detailed and analyzed. We established and validated a nomogram model for predicting childhood NAFLD. Among which, 3036 cases were used as the training set and 180 cases were used as the validation set.

A retrospective study was conducted among 4276 children with obesity who presented for metabolic assessment from January 2003 to May 2021 at The Children’s Hospital of Zhejiang University. Children with BMI exceeding the 95th percentile of their age were diagnosed as obese, and BMI was calculated by dividing weight (kg) by height squared (m²). Exclusion criteria (1): participants missing anthropometric, laboratory, and image data (2); participants with systemic or organic diseases (3); participants with chronic liver disease caused by other known reasons (3); participants taking antihypertensive drugs, antidiabetic drugs, lipid-lowering drugs, uric acid-lowering drugs, or those taking hepatotoxic agents. Finally, 3216 obese children (aged from 2 to 18 years) were enrolled in this study, among which, 3036 cases were used as the training set for the establishment of the predictive model for NAFLD. The remaining180 cases who underwent liver ^1^H-MRS were used as the validation set. This study was approved by the Ethics Committee of Zhejiang University (China). All patients and their families gave informed consent. The flowchart is shown in **Figure 1**.

### Anthropometric data collection and measurement

All of the patients underwent detailed history and physical examinations by trained pediatric endocrinologists. Blood pressure was measured on the right upper arm in a sitting position after a ten-minute rest. The recommended criteria for hypertension in children are as follows: the systolic blood pressure (SBP) of infants > 100/60 mmHg, preschool children > 110/70 mmHg, school-age children > 120/80 mmHg, and those over 13 years old > 140/90 mmHg (25). The subjects were measured for height and weight without shoes. WC was measured in a standing position between the lowest costal margin and the top of the iliac crest. HC was measured at the horizontal level of the widest part of the trochanter. WHtR was calculated as WC/height, and waist-to-hip ratio (WHR) was calculated as WC/HC.

### Laboratory and image data collection and measurement

Fasting blood samples were collected for metabolic assessment. TC, triglycerides (TG), low-density lipoprotein (LDL), high-density lipoprotein cholesterol (HDL-C), apolipoprotein A1(ApoA1), apolipoprotein B (ApoB), uric acid, aminotransferase (AST), and alanine aminotransferase (ALT) were measured by routine laboratory testing (Synchron Clinical System CX4; Beckman Instruments, Columbia, Maryland). Hemoglobin A1c (HbA1c) was measured by high-performance liquid chromatography. All participants underwent a standard oral glucose tolerance test (OGTT), and blood glucose and insulin levels were measured every 30 minutes from 0 min to 120 min during OGTT. Glucose levels were measured by the standard glucose oxidase method on a glucose analyzer (Beijing North Institute of Biological Technology, Beijing, China). Plasma levels of insulin were determined by radioimmunoassay (Beijing North Institute of Biological Technology, Beijing, China).

Insulin sensitivity index (ISI): ISI (composite) was calculated as k/sqrt (G0×I0×G120×I120). G0 and G120 represent the blood glucose level at 0 and 120 min, I0 and I120 represent the insulin level at 0 and 120 min of OGTT (26).

HOMA-IR = fasting glucose level (mmol/L) × fasting insulin level (μU/mL)/22.5 (27). The triglyceride−glucose index (TyG-index) = ln [fasting triglyceride level (mg/dL) × fasting glucose level (mg/dL)/2] (28). TyG-WC, TyG-BMI, and TyG-WHtR were calculated as TyG-index × WC, TyG-index × BMI, and TyG-index × WHtR, respectively (29). TG/HDL-C ratio was defined as TG (mmol/L)/HDL-C (mmol/L) (30).

Liver B-ultrasound was performed by trained sonographers on all participants (Philips 5500). All the participants received B-ultrasound. Of these, 3026 cases were used as the training set to establish the prediction model for NAFLD. The 180 cases that received additional hepatic ^1^H-MRS were used as the validation set. Hepatic ^1^H-MRS using a 1.5-T clinical MR imager while the patient was at rest and supine (Magnetom Avanto, Siemens Healthcare, Erlangen, Germany) (31). Relative liver lipid content was obtained using the equations verified by Longo et al (32). ^1^H-MRS is comparable to liver biopsy in liver fat quantification, is relatively noninvasive, and can even avoid sampling error.

### Diagnosis criteria

NAFLD was diagnosed according to the Chinese consensus on the diagnosis and treatment of NAFLD in children released by the Chinese Society of Pediatric Endocrinology and Metabolism (33). NAFL is diagnosed if a patient meets 1 and 3 of the following criteria; NASH is clinically diagnosed if a patient meets 1-3 of the following criteria (1): chronic hepatic steatosis in children under 18 years old, which is not secondary to an infection, genetic diseases, alcohol consumption, drugs, or malnutrition (2); serum ALT level was greater than 60U/L and lasted more than 3 months in the condition of lifestyle intervention (3); imaging findings meet the diagnostic criteria for diffuse fatty liver disease.

Diagnostic criteria for diabetes mellitus were according to the American Diabetes Association 2019 (34). Criteria for diabetes mellitus (1): fasting plasma glucose (FPG) ≥ 126 mg/dL (7.0 mmol/L), fasting is defined as no caloric intake for at least 8 h (2). 2-h plasma glucose (2-h PG) ≥ 200 mg/dL (11.1 mmol/L) during a 75-g OGTT (3), A1C ≥ 6.5% (4), In a patient with classic symptoms of hyperglycemia or hyperglycemic crisis, a random plasma glucose ≥ 200 mg/dL (11.1 mmol/L). And T2DM was diagnosed by insulin C peptide release assay. IFG: FPG between 5.6 and 6.9 mmol/L, IGT: 2-h PG between 7.8 and 11.0 mmol/L.

MetS in children aged between 6 and 18 years was defined as central obesity (defined as waist circumference larger than 90th percentile) and at least two of the following MetS components (1): IFG: FPG > 5.6 mmol/L or T2DM (2), systolic or diastolic blood pressure ≥ 95th percentile of blood pressure in children of the same age and sex (3), high serum TG level ≥ 150 mg/dL (1.7 mmol/L), or (4) decreased serum HDL-C <40 mg/dL (1.03 mmol/L) (35, 36).

Dyslipidemia included (1): hypertriglyceridemia: TG ≥ 1.76mmol/L (2), hypercholesterolemia: TC ≥ 5.2mmol/L (3), low high-density lipoproteinemia: HDL-C ≤ 1.03mmol/L, (4) high low-density lipoproteinemia: LDL-C ≥ 3.38mmol/L (37).

Hyperuricemia in children was defined as follows: the serum uric acid level > 500 μmol/L (1 to 12 months), > 320 μmol/L (1 to 10y), > 470 μmol/L (11 to 15y males), > 350 μmol/L (11 to 15y females), > 420 μmol/L (males over 15y and menopausal females), > 360 μmol/L (non-menopausal females over 15y) (38, 39).

### Statistical analysis

Counts (percentages) were presented for categorical variables. For continuous variables, means ± SD represented normal distribution data, and quartile intervals represented non-normally distributed data. The significance of differences between groups was evaluated by the Cochran-Armitage test for continuous variables and the chi-squared test for categorical variables. We performed multinomial logistic regression to assess the association between quartiles of indexes for lipid and glucose metabolic disorders [ISI (composite), TyG, TyG-BMI, TyG-WC, TyG-WHtR, TG-HDL, HOMA-IR] and NAFLD with three outcomes (obese without NAFLD, NAFL, NASH). Tests for linear trends were conducted by entering the median value of predictors in each category as a continuous variable in the models. Univariate analyses were performed to determine the factors associated with NAFLD. Linear regression and colinear diagnosis were performed to exclude colinear factors and variance inflation factors (VIF) of >10 indicated collinearity between variables. A stepwise multivariate logistic regression was conducted to identify independent predictors of NAFLD and estimate the odds ratio (OR) and 95% confidence interval (CI). Cofounding variables including age, BMI, SBP, DBP, HOMA-IR, LDL-C, Lipid protein A, Apo B, AST, TC, TG, uric acid, 2h glucose, and fasting glucose were adjusted. A nomogram composed of eight parameters was conducted for the prediction of a patient’s probability of having NAFLD. Area under the receiver operating characteristics curve (AUROC) analysis, sensitivity, and specificity were used to assess the accuracy of the model. SPSS 26.0 and R software (version 4.0.2) were used for statistical analyses and a P value <0.05 was considered statistically significant.

## Results

### Metabolic spectrum of children with obesity and comorbidities of pediatric NAFLD

**Figure 2:**
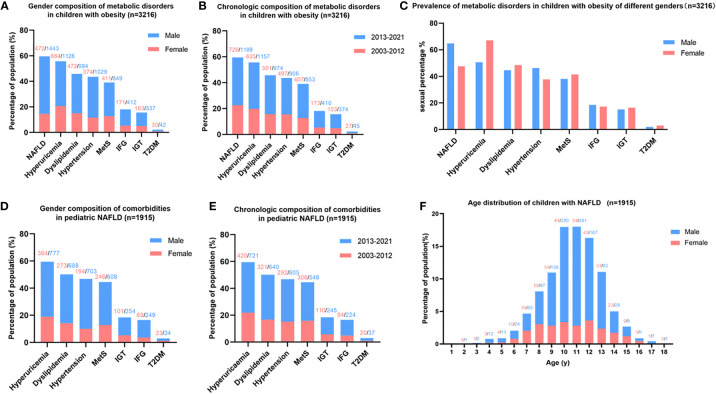
Metabolic spectrum of childhood obesity and comorbidities of pediatric NAFLD. **(A)** Gender composition of metabolic disorders in children with obesity: NAFLD is the most common complication of childhood obesity, more males suffered from obesity and metabolic disorder than females. **(B)** Chronologic composition of metabolic disorders in children with obesity: The prevalence of obesity and metabolic disorders in children has been increasing in recent 19 years. **(C)** Prevalence of metabolic disorders in children with obesity adjusted by gender: the prevalence of NAFLD and hypertension was much higher in males than females, while hyperuricemia was much more prevalent in females than males. **(D)** Gender composition of comorbidities in pediatric NAFLD: hyperuricemia ranked top one comorbidities of childhood NAFLD. **(E)** Chronologic composition of comorbidities in pediatric NAFLD: The prevalence of comorbidities of pediatric NAFLD has been increasing in the last 19 years. **(F)** Age distribution of children with NAFLD: the peak onset age of childhood NAFLD is 10-12 years old. Note: the number on the top of the figure indicates the count of patients, red represents females, and blue represents males.

In total, 3,216 children with obesity aged 2–18.0 years were enrolled in this study. Of these, 1915 cases (59.55%) were NAFLD by B-ultrasound, among which, 1,443 were males (75.35%) and 472 were females (24.65%). A metabolic assessment revealed that NAFLD was the most common complication of childhood obesity, followed by hyperuricemia, dyslipidemia, hypertension, metabolic syndrome, IFG, IGT, and T2DM (**Figure 2A**). The prevalence of obesity and metabolic disorders has been increasing over the last 19 years (**Figure 2B**). The prevalence of NAFLD and hypertension was much higher in males, while the prevalence of hyperuricemia, dyslipidemia, and metabolic syndrome (MetS) was higher in females. The prevalence of dysglycemia was almost equal in both genders (**Figure 2C**). The top comorbidity of NAFLD in children was hyperuricemia, followed by dyslipidemia, hypertension, MetS, IGT, IFG, and T2DM (**Figures 2D, E**). More males suffered from these comorbidities than females (**Figure 2D**), and the prevalence of comorbidities among pediatric NAFLD has been increasing over the last 19 years (**Figure 2E**). The onset age of NAFLD in children was normally distributed, and the peak onset age was 10-12 years old (**Figure 2F**).

### Characteristics of pediatric NAFLD

The clinical features of 3,216 children with obesity were detailed and analyzed according to different time periods (**Table 1**) and genders (**Table 2**). They were further divided into three sub-groups according to the Chinese consensus on the diagnosis and treatment of NAFLD in children: obese without NAFLD, NAFL, and NASH groups. Linear trend analysis revealed that BMI, height, weight, WC, HC, fasting insulin, 2h insulin, TC, and uric acid were linearly correlated with NAFLD regardless of time or gender (p < 0.05). Age was linearly correlated with NAFLD in both 2003~2012 and 2013~2021, and also correlated with NAFLD in females but had no correlation with NAFLD in males. WHtR was linearly correlated with NAFLD in both genders, and also linearly correlated with NAFLD in 2013–2021 but had no correlation in 2003–2012. ALT was linearly correlated with NAFLD in patients from 2013–2021 and females, but had no correlation in patients from 2003–2012 and males. TG was linearly correlated with NAFLD in females only (**Tables 1**, **2**).

**Table 1:** Clinical features of the participants over the recent 19 years.

	Total	2003-2012				2013-2021			
		Obese without NAFLD	NAFL	NASH	P valuefor trend	Obese without NAFLD	NAFL	NASH	P valuefor trend
n	3216	456 (14.18%)	430 (13.37%)	296 (9.20%)		845 (26.27%)	834 (25.93%)	355 (11.04%)	
female	991	181 (18.26%)	125 (12.61%)	53 (5.35%)		338 (34.11%)	231 (23.31%)	63 (6.36%)	
male	2225	275 (12.36%)	305 (13.71%)	243 (10.92%)	0.0029	507 (22.79%)	603 (27.10%)	292 (13.12%)	2.20E-16
Age (y)	10.18 ± 2.53	9.11 ± 2.70	10.30 ± 2.36	10.64 ± 2.24	0.0107	9.66 ± 2.68	10.61 ± 2.33	11.26 ± 1.99	0.0078
Anthropometry									
BMI (kg/cm^2^)	28.27 ± 4.19	26.45 ± 3.40	28.61 ± 4.15	29.01 ± 3.88	7.00E-04	26.76 ± 3.78	29.53 ± 4.06	30.23 ± 4.53	2.20E-16
Weight (kg)	63.58± 18.76	53.60 ± 15.84	64.82 ± 17.23	66.59 ± 16.29	3.00E-04	57.62 ± 17.98	69.00 ± 18.21	73.83 ± 18.60	2.20E-16
Height (cm)	148.21 ± 14.64	140.75 ± 15.54	149.20 ± 12.84	150.42 ± 12.06	0.0019	144.79 ± 15.38	151.55 ± 13.47	155.07 ± 12.04	4.00E-04
WC (cm)	89.37 ± 11.40	83.23 ± 9.56	90.54 ± 10.45	92.04 ± 10.08	2.00E-04	84.89 ± 10.74	92.90 ± 10.70	95.98 ± 11.14	2.20E-16
HC (cm)	94.83 ± 11.31	89.06 ± 10.44	95.07 ± 10.62	95.44 ± 9.48	0.0026	91.75 ± 11.21	98.62 ± 10.62	99.85 ± 10.95	2.20E-16
WHtR	0.62 ± 0.09	0.59 ± 0.06	0.61 ± 0.05	0.61 ± 0.05	0.2012	0.59 ± 0.06	0.65 ± 0.11	0.65 ± 0.11	2.20E-16
WHR	0.93 ± 0.09	0.94 ± 0.06	0.96 ± 0.07	0.97 ± 0.06	0.2983	0.92 ± 0.08	0.91 ± 0.12	0.93 ± 0.12	0.5788
Blood pressure									
SBP (mmHg)	120.34 ± 14.14	111.99 ± 13.32	116.86 ± 14.39	119.77 ± 14.66	0.0665	120.66 ± 13.35	123.81 ± 13.03	126.91 ± 12.49	0.1528
DBP (mmHg)	69.94 ± 9.48	67.05 ± 8.44	68.60 ± 8.93	69.54 ± 7.89	0.4069	69.71 ± 9.95	71.13 ± 9.66	73.39 ± 9.65	0.385
Glucose metabolism									
HbA1c (%)	5.77 ± 0.57	5.76 ± 0.48	5.87 ± 0.51	5.87 ± 0.54	0.3674	5.67 ± 0.51	5.74 ± 0.60	5.88 ± 0.74	0.3796
Fasting glucose (mmol/L)	5.35 ± 0.62	5.25 ± 0.58	5.22 ± 0.61	5.29 ± 0.68	0.795	5.37 ± 0.48	5.38 ± 0.54	5.54 ± 0.93	0.9156
Fasting insulin (uIU/ml)	21.96 ± 16.93	16.11 ± 11.99	20.70 ± 14.87	22.38 ± 19.52	0.0214	20.71 ± 16.50	24.47 ± 17.91	27.69 ± 18.16	0.0086
2h glucose (mmol/L)	6.83 ± 1.47	6.50 ± 1.02	6.60 ± 1.18	7.11 ± 1.79	0.6615	6.64 ± 1.12	6.90 ± 1.45	7.57 ± 2.28	0.1524
2h insulin (uIU/ml)	89.20 ± 95.41	38.53 ± 47.78	75.42 ± 76.02	91.28 ± 86.46	2.20E-16	89.59 ± 102.84	105.53 ± 97.47	129.94 ± 114.47	0.0077
Lipid profile									
TG (mmol/L)	1.38 ± 0.88	1.32 ± 1.27	1.40 ± 0.74	1.56 ± 0.76	0.1466	1.26 ± 0.67	1.37 ± 0.81	1.54 ± 1.03	0.1102
TC (mmol/L)	4.29 ± 0.86	4.29 ± 0.85	4.28 ± 0.80	4.51 ± 0.85	0.9949	4.19 ± 0.82	4.25 ± 0.91	4.49 ± 0.90	0.7265
LDL-C (mmol/L)	2.57 ± 0.66	2.40 ± 0.63	2.42 ± 0.65	2.60 ± 0.68	0.8325	2.56 ± 0.59	2.64 ± 0.67	2.81 ± 0.70	0.3487
HDL-C (mmol/L)	1.22 ± 0.27	1.26 ± 0.32	1.20 ± 0.29	1.14 ± 0.25	0.2954	1.25 ± 0.27	1.21 ± 0.25	1.21 ± 0.26	0.2582
Inflammatory marker									
Uric acid (umol/L)	390.23 ± 96.51	349.72 ± 81.74	382.94 ± 95.53	418.90 ± 91.92	0.021	371.01 ± 91.46	401.51 ± 94.88	446.49 ± 97.75	0.0026
AST (U/L)	34.97 ± 25.38	27.22 ± 13.85	27.73 ± 9.56	66.42 ± 35.77	0.7952	25.87 ± 11.76	26.80 ± 9.19	68.33 ± 38.22	0.5436
ALT (U/L)	46.83 ± 47.64	26.12 ± 18.73	32.89 ± 13.28	116.26 ± 60.95	0.1345	25.94 ± 19.57	31.79 ± 13.40	117.51 ± 64.13	0.0344

**Table 2:** Clinical features of the participants grouped by gender.

	Total	Male				Female			
		Obese without NAFLD	NAFL	NASH	P valuefor trend	Obese without NAFLD	NAFL	NASH	P valuefor trend
n	3216	782 (24.32%)	908 (28.23%)	535 (16.64%)	2.20E-16	519 (16.14%)	356 (11.07%)	116 (3.61%)	2.20E-16
Age (y)	10.18 ± 2.53	9.82 ± 2.66	10.52 ± 2.21	11.04 ± 2.04	0.063	8.94 ± 2.68	10.45 ± 2.65	10.73 ± 2.51	0.002
Anthropometry									
BMI (kg/cm^2^)	28.27 ± 4.19	27.20 ± 3.26	29.24 ± 3.80	29.73 ± 4.18	2.20E-16	25.83 ± 4.03	29.16 ± 4.84	29.47 ± 4.74	2.20E-16
Weight (kg)	63.58± 18.76	58.88 ± 16.95	68.17 ± 17.32	71.28 ± 17.54	1.00E-04	52.18 ± 17.21	66.07 ± 19.51	67.10 ± 19.42	2.20E-16
Height (cm)	148.21 ± 14.63	145.53 ± 15.86	151.48 ± 13.22	153.77 ± 12.05	0.0047	140.13 ± 14.49	148.89 ± 13.36	149.20 ± 12.58	3.00E-04
WC (cm)	89.37 ± 11.40	86.84 ± 9.61	93.14 ± 10.07	94.99 ± 10.36	2.20E-16	80.50 ± 10.32	89.44 ± 11.67	90.51 ± 12.24	2.20E-16
HC (cm)	94.83 ± 11.31	91.99 ± 10.10	97.23 ± 10.08	97.96 ± 10.11	6.00E-04	89.02 ± 12.07	97.86 ± 12.30	97.30 ± 12.32	2.20E-16
WHtR	0.62 ± 0.09	0.60 ± 0.06	0.64 ± 0.10	0.64 ± 0.10	9.00E-04	0.58 ± 0.06	0.62 ± 0.10	0.61 ± 0.08	8.00E-04
WHR	0.93 ± 0.09	0.94 ± 0.07	0.94 ± 0.11	0.95 ± 0.10	0.8792	0.91 ± 0.07	0.89 ± 0.11	0.92 ± 0.09	0.7412
Blood pressure									
SBP (mmHg)	120.34 ± 14.14	118.78 ± 14.34	121.70 ± 13.77	124.27 ± 13.89	0.1784	115.84 ± 13.20	120.77 ± 14.23	120.78 ± 14.03	0.0723
DBP (mmHg)	69.94 ± 9.48	69.26 ± 9.40	70.11 ± 9.35	71.56 ± 9.07	0.5852	68.04 ± 9.69	70.68 ± 9.84	71.97 ± 9.20	0.1863
Glucose metabolism									
HbA1c (%)	5.77 ± 0.57	5.73 ± 0.52	5.79 ± 0.58	5.86 ± 0.64	0.5246	5.65 ± 0.47	5.77 ± 0.55	5.95 ± 0.75	0.2329
Fasting glucose (mmol/L)	5.35 ± 0.62	5.33 ± 0.49	5.33 ± 0.51	5.43 ± 0.83	0.9776	5.33 ± 0.57	5.32 ± 0.70	5.41 ± 0.84	8.68E-01
Fasting insulin (uIU/ml)	21.96 ± 16.93	19.41 ± 16.24	21.88 ± 15.68	24.29 ± 17.41	0.0472	18.62 ± 13.56	26.52 ± 19.69	29.83 ± 24.47	6.00E-04
2h glucose (mmol/L)	6.83 ± 1.47	6.58 ± 1.03	6.70 ± 1.27	7.23 ± 1.97	0.5127	6.60 ± 1.16	7.05 ± 1.58	7.98 ± 2.45	6.43E-02
2h insulin (uIU/ml)	89.20 ± 95.41	74.56 ± 91.05	89.01 ± 89.20	108.45 ± 99.16	0.013	67.38 ± 90.54	111.30 ± 96.51	130.40 ± 124.73	2.20E-16
Lipid profile									
TG (mmol/L)	1.38 ± 0.88	1.27 ± 0.67	1.35 ± 0.83	1.51 ± 0.92	0.3497	1.31 ± 1.21	1.48 ± 0.68	1.75 ± 0.89	0.0072
TC (mmol/L)	4.29 ± 0.86	4.28 ± 0.79	4.26 ± 0.84	4.51 ± 0.87	0.7759	4.15 ± 0.87	4.26 ± 0.94	4.45 ± 0.93	4.38E-01
LDL-C (mmol/L)	2.57 ± 0.66	2.52 ± 0.59	2.54 ± 0.66	2.71 ± 0.69	0.8233	2.47 ± 0.64	2.62 ± 0.69	2.75 ± 0.73	0.1721
HDL-C (mmol/L)	1.22 ± 0.27	1.27 ± 0.29	1.22 ± 0.27	1.20 ± 0.26	0.2074	1.22 ± 0.28	1.17 ± 0.24	1.08 ± 0.23	2.52E-01
Inflammatory marker									
Uric acid (umol/L)	390.23 ± 96.51	370.38 ± 91.96	399.12 ± 98.85	434.27 ± 97.14	0.0102	353.33 ± 82.68	385.19 ± 85.54	432.92 ± 91.37	0.005
AST (U/L)	34.97 ± 25.38	27.12 ± 12.57	27.73 ± 9.40	68.46 ± 37.55	0.7122	25.18 ± 12.41	25.56 ± 8.95	62.84 ± 34.78	0.7511
ALT (U/L)	46.83 ± 47.64	27.75 ± 20.17	33.36 ± 13.16	120.35 ± 65.44	0.1048	23.37 ± 17.51	29.13 ± 13.42	101.23 ± 44.75	0.0448

Clinical features of the participants over the last 19 years revealed that the prevalence of obesity, with and without NAFLD, increased during 2013–2021 when compared with 2003–2012. BMI, WC, HC, SBP, DBP, fasting insulin, 2h insulin, LDL-C, and uric acid in each group also increased significantly during 2013–2021 when compared with 2003–2012. Interestingly, WHtR did not change in the obese without NAFLD group but increased significantly in the NAFL and NASH group during 2013–2021 compared with 2003–2012 (**Table 1**).

Clinical features of the participants of different genders revealed that obese children with and without NAFLD were more common in males than females. WC, WHtR, WHR, SBP, fasting insulin, 2h insulin, HDL-C, ALT, and AST were much higher in males than females in each group. While 2h glucose and TG level were much higher in females than males in each group (**Table 2**).

### Cardiometabolic risk factors of pediatric NAFLD

Cardiometabolic risk factors of pediatric NAFLD, including ISI (composite), HOMA-IR, TyG-index, TyG-BMI, TyG-WC, TyG-WHtR, and TG/HDL-C, were divided into four groups (Q1, Q2, Q3, and Q4) according to the quartile loci of these indicators (**Supplemental Table S1**). Linear trend analysis showed that all the indicators were linearly correlated with pediatric NAFLD (p < 0.05, **Supplemental Table S2**). Multivariate regression analysis revealed that five of the cardiometabolic risk factors, including ISI (composite), HOMA-IR, TyG-BMI, TyG-WC, and TyG-WHtR, were significant indicators associated with NAFLD when adjusted for age and gender (p < 0.05), while TyG-index and TG/HDL-C were not significantly associated with pediatric NAFLD (**Table 3**). TyG-WC was the most significant factor associated with NAFL (adjusted odds ratio = 5.74, 95% CI: 4.30 - 7.67, p < 0.05) and NASH (adjusted odds ratio = 10.23, 95% CI: 7.00 - 14.95, p < 0.05).

**Table 3:** Cardiometabolic risk factors odds Ratio for NAFLD adjusted for age and sex.

Cardiometabolic risk factors	group	NAFL	NASH	p for trend
ISI (composite)	Q2	0.74 (0.59-0.94)	0.53 (0.41-0.7)	5.31E-13
	Q3	0.58 (0.46-0.74)	0.31 (0.23-0.41)	
	Q4	0.41 (0.32-0.52)	0.35 (0.26-0.47)	
HOMA-IR	Q2	1.17 (0.94-1.46)	0.89 (0.67-1.19)	4.31E-03
	Q3	1.58 (1.26-1.98)	1.35 (1.02-1.8)	
	Q4	1.91 (1.51-2.43)	2.11 (1.59-2.81)	
TyG index	Q2	1.07 (0.86-1.33)	1.59 (1.19-2.14)	0.62
	Q3	1.15 (0.93-1.44)	1.66 (1.23-2.23)	
	Q4	1.56 (1.24-1.96)	3.13 (2.35-4.19)	
TyG-BMI	Q2	1.64 (1.3-2.06)	1.86 (1.36-2.54)	0.01
	Q3	2.57 (2.03-3.25)	2.6 (1.89-3.59)	
	Q4	4.2 (3.23-5.48)	6.22 (4.46-8.67)	
TyG-WC	Q2	2.14 (1.69-2.7)	2.75 (1.95-3.88)	4.73E-2
	Q3	3.23 (2.5-4.16)	4.37 (3.07-6.24)	
	Q4	5.74 (4.3-7.67)	10.23 (7-14.95)	
TyG-WHtR	Q2	1.74 (1.39-2.18)	2.3 (1.69-3.13)	3.11E-04
	Q3	2.19 (1.74-2.76)	3.34 (2.46-4.53)	
	Q4	4.26 (3.35-5.42)	6.57 (4.8-8.99)	
TG/HDL-C ratio	Q2	1.2 (0.96-1.5)	1.2 (0.9-1.61)	0.07
	Q3	1.3 (1.04-1.62)	1.54 (1.15-2.05)	
	Q4	1.38 (1.1-1.73)	2.36 (1.79-3.12)	

### Establishment and validation of a clinical prediction model for pediatric NAFLD

**Figure 3:**
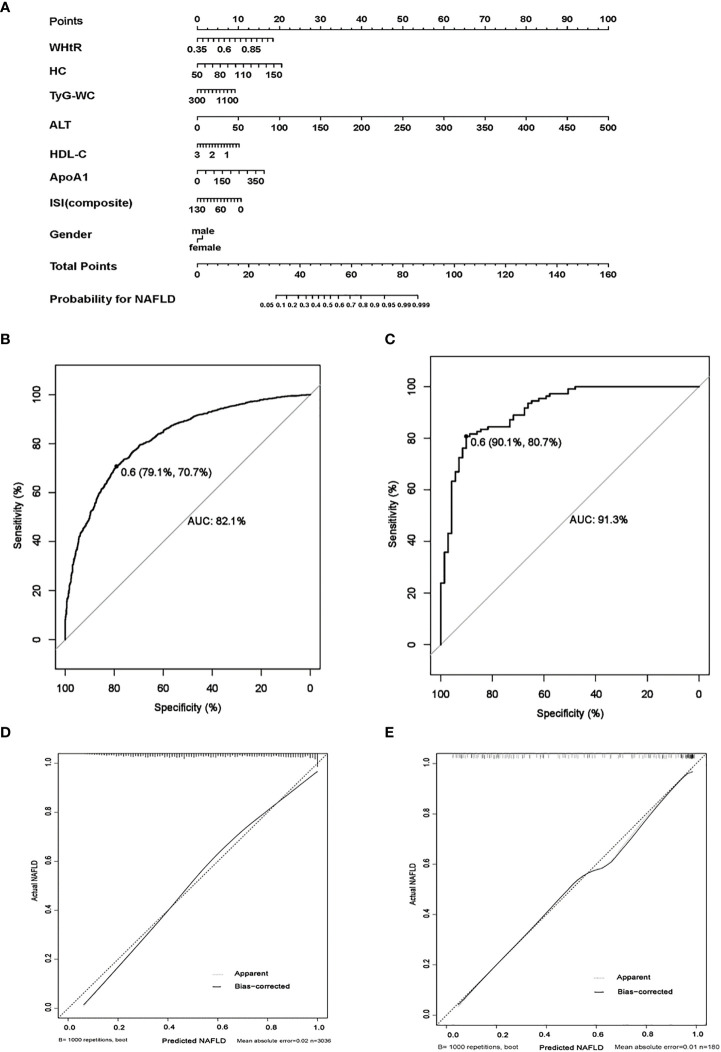
The establishment and validation of a clinical predicting model for childhood NAFLD. **(A)** Nomogram model for predicting childhood NAFLD. **(B)** Receiver operating characteristics (ROC) curves for predicting NAFLD in the training set, the AUROC of the model is 0.821 (95% CI 0.806–0.835, p < 0.001) with the sensitivity and specificity of 70.70% and 79.10%, respectively. **(C)** ROC curves for predicting NAFLD in the validation set, the AUROC of the model is 0.913 (95% CI 0.879–0.960, p < 0.001) with the sensitivity and specificity of 80.70% and 90.10%, respectively. **(D, E)**: calibration curves for assessment of the performance of the nomogram in the training set **(D)** and the validation set **(E)**. (1000 bootstrap resamples). Nomogram predicted probability of NAFLD is plotted on the x-axis; actual probability is plotted on the y-axis.

The clinical characteristics of the training and validation set are shown in **Supplemental Table S3**. After a stepwise multivariate logistic regression, we established a nomogram for predicting NAFLD based on anthropometric and laboratory indicators (**Figure 3A**). Eight variables including WHtR, HC, TyG-WC, ALT, HDL-C, ApoA1, ISI (composite), and gender were chosen to establish the predicting model. The variables in the equation are shown in **Supplementary Table S4**. Each variable was assigned a score from 0 to 100, among which, ALT scored the highest. A total score was calculated by each covariate and then placed on the total point scale. In this way, the possibility of NAFLD could be efficiently estimated. The AUROC of the prediction model was 0.821 (95% CI 0.806–0.835, p < 0.001), and, with a cut-off value of 0.60, the sensitivity and specificity were 70.70% and 79.10%, respectively (**Figure 3B**).

We also established a nomogram for predicting NAFLD in males and females respectively. The AUROC of the six-parameter nomogram model including WHtR, HC, ALT, HDL-C, ApoA1, and ISI (composite) for predicting male NAFLD was 0.817 (95% CI 0.798–0.835, p < 0.001). The AUROC of the seven-parameter nomogram model including WHtR, age, TyG-WC, ALT, TC, ApoB, and HOMA-IR for predicting female NAFLD was 0.810 (95% CI 0.782–0.837, p < 0.001) (**Supplementary Figure S1**).

The performance of gender-independent predictive models was not better than the eight-parameter nanogram. Thus, liver fat content detected by ^1^H-MRS was used as the gold standard of NAFLD to validate the efficiency of the eight-parameter nomogram model. The prediction model performed well in the validation set, and the AUROC was 0.913 (95% CI 0.879–0.960, p < 0.001), with a cut-off value of 0.60. The sensitivity and specificity were 80.70% and 90.10%, respectively (**Figure 3C**). The calibration curve of the prediction model of pediatric NAFLD was extremely close to the diagonal line and demonstrated agreement between the training and validation sets (**Figures 3D**, **E**).

## Discussion

The prevalence of pediatric NAFLD is projected to continually increase, considering the change in lifestyle and the ongoing obesity epidemic beginning in childhood. However, the epidemiology, natural history, and clinical spectrum of childhood NAFLD are not clarified due to the lack of efficient and cost-effective assessment approaches. This study obtained a large sample database of children with obesity (1,264 NAFL, 651 NASH, and 1,301 obese without NAFLD), and detailed the clinical spectrum, chronologic and gender differences, and cardiometabolic risk factors of NAFLD. Further, this study established and validated a predictive model of childhood NAFLD based on anthropometric and laboratory indicators. These data enriched the metabolic spectrum of childhood NAFLD and played an important role in screening, follow-up, and evaluation of pediatric NAFLD.

In this study, NAFLD is the most common complication among children with obesity, followed by hyperuricemia, dyslipidemia, hypertension, MetS, and dysglycemia. The prevalence of NAFLD in China has increased in parallel with childhood obesity over the previous 19 years, which may be the result of rapid economic development, changes in lifestyle, and diet patterns. 59.55% of obese children were diagnosed with NAFLD, which was equal to that of Western developed countries (40). Cardiometabolic indexes such as BMI, WC, HC, SBP, DBP, fasting insulin, 2h insulin, LDL-C, and uric acid increased significantly over the last 19 years. Interestingly, the WHtR did not change in the obese without NAFLD group but increased significantly in the NAFL and NASH groups over the same time period, indicating the vital role of WHtR in predicting childhood NAFLD.

Males were more likely to develop NAFLD, and the youngest pediatric NAFLD patient was a two-year-old boy. The gender differences in the prevalence of NAFLD may be explained by fat distribution and the protective effect of estrogen. Studies revealed that estrogen may improve insulin sensitivity, lower liver *de novo* lipogenesis, reduce triglyceride accumulation, and ameliorate liver fibrosis and inflammation (41–44). The peak onset age of NAFLD is 10–12 years old. This may be related to exposure to an unhealthy lifestyle and, more importantly, the onset of puberty, where insulin resistance may increase the incidence of NAFLD. Literature has reported that insulin resistance rises progressively from the age of 7, with a peak age of 12 for females, while rose slowly and tended to be stabilized in males after 10 years old (45). Considering the majority of NAFLD were boys, testosterone levels rise with sexual maturity, and higher testosterone was correlated with improved features of steatosis and fibrosis (46). These data explain our results that showed that the prevalence of NAFLD rose from the age of 6, peaked at 10–12 years, and then began to decline. Thus, lifestyle interventions, as well as psychological support, should be prescribed. In case of failure, metformin can be used in children aged 10 or more, which has been shown to be beneficial to patients with NAFLD (47).

Hyperuricemia ranked as the most common comorbidity among those with childhood NAFLD (59.58%), followed by dyslipidemia, hypertension, MetS, IGT, IFG, and T2DM. The high incidence of hyperuricemia may be attributed to the diet pattern of children. A dietary survey in the US showed that fructose consumption was much higher in children and adolescents than in adults, and the largest source of fructose was sugar-sweetened beverages (48). The study revealed that fructose consumption was the only factor independently associated with serum uric acid, and both fructose consumption and serum uric acid were independently associated with NASH in children and adolescents. Fructose can generate uric acid during its metabolism in the liver, stimulate *de novo* lipogenesis, and trigger inflammation (49, 50). A recent prospective cohort study showed that the increased incidence of NAFLD among obese people seemed to be affected by obesity-related changes in serum uric acid (51). Thus, serum uric acid can be used to evaluate the progression of NAFLD, and controlling fructose intake and promoting uric acid excretion is of great value for the prevention and treatment of pediatric NAFLD.

Although most children are in this study are in the NAFL group, a clinically silent disease that generally goes undiagnosed, a study has shown that young children are more likely to progress to NASH and cirrhosis than adults, which may be related to the higher activity of the hedgehog signaling pathway (52). However, few children have regular physical examinations, especially liver ultrasounds, and liver biopsy performed on obese children is extremely rare. Thus, a rapid, accurate, and noninvasive model for predicting NAFLD is urgently needed in pediatrics. Studies have reported that lipid profiles, machine learning models based on liver ultrasound images, ALT/AST ratio, and anthropometric data were powerful and easy tools to predict childhood NAFLD (53–56). However, the sample size of the first two studies was limited, and the sensitivity and specificity of a single indicator in predicting NAFLD still need to be improved. We confirmed that ISI (composite), HOMA-IR, TyG-index, TyG-BMI, TyG-WC, TyG-WHtR, and TG/HDL-C were linearly correlated with pediatric NAFLD. Of these, TyG-WC was the most significant factor associated with NAFL and NASH with an odds ratio of 5.74 and 10.23, respectively. After a stepwise multivariate logistic regression, an eight-parameter nomogram model including WHtR, HC, TyG-WC, ALT, HDL-C, ApoA1, ISI (composite), and gender was established and validated to have a better potential for predicting NAFLD, with an AUROC of 0.913 (sensitivity 80.70%, specificity 90.10%).

This nomogram model was specific to children, developed from a large data sample, with high accuracy, and satisfactory sensitivity and specificity. It provided a visualized approach for pediatricians, especially for community medical staff, to quickly screen those at high risk of NAFLD. However, there are still several limitations. First, the diagnosis and grouping of NAFLD were based on liver B-ultrasound, and the nomogram model may potentially miss a diagnosis of NAFLD due to the poor sensitivity of ultrasound in mild steatosis patients. As such, we adopted liver ^1^H-MRS as verification. Studies showed that ^1^H-MRS is comparable to liver biopsy in liver fat quantification, is relatively noninvasive, and can even avoid sampling error (57, 58). Second, this is a single-center, retrospective study, and the enrolled subjects were children with obesity, who cannot reflect the overall prevalence of childhood NAFLD. However, the majority of pediatric NAFLD cases are obesity-related. Studies on the prevalence of pediatric NAFLD in the population have shown that the prevalence of NAFLD ranged from 40% to 70% in obese children, compared to just 3% to 11% in healthy children (4–8). The high prevalence of NAFLD in obese children and the high prevalence of hyperuricemia in pediatric NAFLD suggested that controlling weight, reducing fructose consumption, and increasing uric acid excretion were important factors in the prevention and treatment of pediatric NAFLD. A multicenter, prospective study including healthy children is needed to further describe the epidemiology, natural history, and clinical spectrum of pediatric NAFLD, and optimize the rapid diagnosis model of childhood NAFLD in the general population.

## Conclusion

In this study, we found that NAFLD was the most common complication of obesity and hyperuricemia was the first comorbidity of pediatric NAFLD. Males were more likely to suffer from NAFLD and the peak onset age was 10–12 years old. An eight-parameter nomogram model was confirmed to have a better potential for predicting NAFLD and can be used as a quick screening tool to assess pediatric NAFLD in children with obesity.

## Data availability statement

The original contributions presented in the study are included in the article/**Supplementary Material**. Further inquiries can be directed to the corresponding author.

## Ethics statement

This study was approved by the Ethics Committee of Zhejiang University (China). Written informed consent to participate in this study was provided by the participants’ legal guardian/next of kin.

## Author contributions

XZ, XL, and JF had full access to all the data in the study and take responsibility for the integrity of the data and the accuracy of the data analysis. Concept and design: JF, XZ, XL. Acquisition, analysis, or interpretation of data: All authors. Drafting of the manuscript: XZ, XL. Data collection: HL, XZ, LZ, YD. Critical revision of the manuscript for important intellectual content: All authors. Statistical analysis: JC, JP, ZW. Administrative, technical, or material support: JF, WW, YN, GD, KH. Supervision: JF, WW, YN, GD, KH. All authors contributed to the article and approved the submitted version.

## Funding

This work was supported by the National Key Research and Development Program of China [2021YFC2701901, 2016YFC1305301], the Zhejiang Medical and Health Science and Technology Project [2022KY870, 2018KY444], National Natural Science Foundation of China [82170583], Zhejiang Province Natural Sciences Foundation Zhejiang Society for Mathematical Medicine [LSZ19H070001], Zhejiang Provincial Key Science and Technology Project [LGF21H070004], the Fundamental Research Funds for the Central Universities [2020XZZX002-22].

## Acknowledgments

We are grateful to our participants and our colleagues who give help to our study.

## Conflict of interest

The authors declare that the research was conducted in the absence of any commercial or financial relationships that could be construed as a potential conflict of interest.

## Publisher’s note

All claims expressed in this article are solely those of the authors and do not necessarily represent those of their affiliated organizations, or those of the publisher, the editors and the reviewers. Any product that may be evaluated in this article, or claim that may be made by its manufacturer, is not guaranteed or endorsed by the publisher.

## Abbreviations

NAFLD, Nonalcoholic fatty liver disease; NAFL, Nonalcoholic fatty liver; NASH, Nonalcoholic steatohepatitis; MetS, Metabolic Syndrome ; T2DM, Type 2 diabetes mellitus ; OGTT, Oral glucose tolerance test; IGT, Impaired glucose tolerance; IFG, Impaired fasting glucose; FPG, Fasting plasma glucose; BMI, Body mass index; WC, Waist circumference; HC, Hip circumference; WHtR, waist-to-height ratio; WHR, waist-to-hip ratio; SBP, Systolic blood pressure; DBP, Diastolic blood pressure; ALT, Alanine aminotransferase; AST, Aspartate aminotransferase; TG, Total triglycerides; TC, Total cholesterol;LDL-C, Low density lipoprotein cholesterol; HDL-C, High density lipoprotein cholesterol; ISI, Insulin sensitivity index; HOMA-IR, Homeostatic model assessment of insulin resistance; TyG-index, Triglyceride−glucose index; TyG-WC, Triglyceride glucose-waist circumference; TyG-BMI, Triglyceride glucose-body mass index; TyG-WHtR, Triglyceride glucose-waist-to-height ratio; ^1^H-MRS, Hydrogen proton magnetic resonance spectroscopy; AUROC, Area under the receiver operator characteristic curve; ApoA1, apolipoprotein A1; ApoB, apolipoprotein B.
