# Supplementary material for: Clinical spectrum transition and prediction model of nonalcoholic fatty liver disease in children with obesity
Source: Front Endocrinol (Lausanne). 2022 Aug 31;13:986841. doi: 10.3389/fendo.2022.986841 (PMC9471666; doi:10.3389/fendo.2022.986841)
Supplement: Supplementary file 1 [file Image_1.pdf]

Supplementary materials

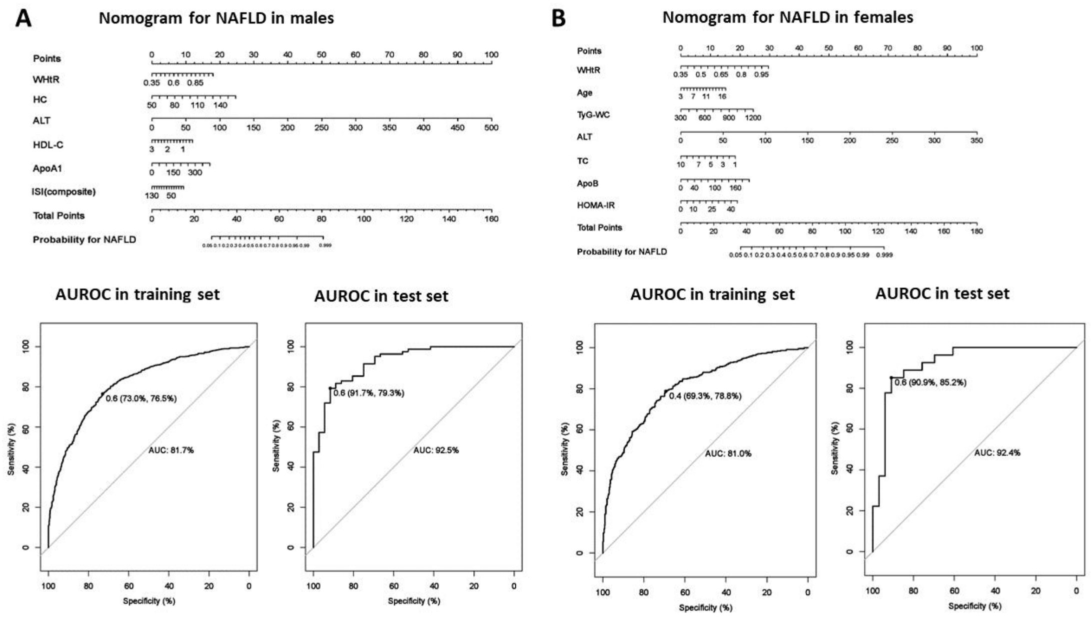

Figure S1 The establishment and validation of clinical predicting model for childhood NAFLD according to different gender. A: A six-parameter Nomogram model including WHtR, HC, ALT, HDL-C, ApoA1 and ISI (composite) for predicting males' NAFLD. The AUROC for predicting NAFLD in training set is 0.817 (95% CI 0.798–0.835, $p < 0.001$), with the sensitivity and specificity of 76.50% and 73.00%, respectively. And the AUROC for predicting NAFLD in validation set is 0.925 (95% CI 0.877–0.973, $p < 0.001$), with the sensitivity and specificity of 79.30% and 91.70%, respectively. B: A seven-parameter Nomogram model including WHtR, age, TyG-WC, ALT, TC, ApoB and HOMA-IR for predicting females' NAFLD. The AUROC for predicting NAFLD in training set is 0.810 (95% CI 0.782–0.837, $p < 0.001$), with the sensitivity and specificity of 78.80% and 69.30%, respectively. And the AUROC for predicting NAFLD in validation set is 0.924 (95% CI 0.855–0.993, $p < 0.001$), with the sensitivity and specificity of 85.20% and 90.90%, respectively.
