# Supplementary material for: Clinical spectrum transition and prediction model of nonalcoholic fatty liver disease in children with obesity
Source: Front Endocrinol (Lausanne). 2022 Aug 31;13:986841. doi: 10.3389/fendo.2022.986841 (PMC9471666; doi:10.3389/fendo.2022.986841)
Supplement: Supplementary file 2 [file DataSheet_1.docx]

**Supplementary materials**

**Table 1 Quartile grouping criteria of Cardiometabolic risk factors among children with obesity**

| **Group** | **ISI (composite)** | | **HOMA-IR** | **TyG index** | **TyG-BMI** | **TyG-WC** | **TyG-WHtR** | **TG/HDL-C ratio** |
| --- | --- | --- | --- | --- | --- | --- | --- | --- |
|  |  | |  |  |  |  |  |  |
| **Q1** | <1.70 | <2.72 | | <8.23 | <213.95 | <686.80 | <4.8 | <1.08 |
| **Q2** | 1.70~2.92 | 2.72~4.37 | | 8.23~8.54 | 213.95~237.91 | 686.80~761.95 | 4.8~5.15 | 1.08~1.45 |
| **Q3** | 2.92~5.84 | 4.37~6.77 | | 8.54~8.85 | 237.91~265.59 | 761.95~833.01 | 5.15~5.57 | 1.45~1.94 |
| **Q4** | ≥5.84 | ≥6.77 | | ≥8.85 | ≥265.59 | ≥833.01 | ≥5.57 | ≥1.94 |

**Table 2 Quartile groups information of cardiometabolic risk factors among children with obesity**

|  |  | **Total** | **Obese without NAFLD** | **NAFL** | **NASH** | **p-value for trend** |
| --- | --- | --- | --- | --- | --- | --- |
| **n** |  | 3216 | 1301 (40.45%) | 1264 (39.31%) | 651(20.24%) |  |
| **ISI (composite)** | **Q1** | 798 | 210 (26.32 %) | 350 (43.86 %) | 238 (29.82 %) | 2.20E-16 |
|  | **Q2** | 809 | 286 (35.35 %) | 350 (43.26%) | 173 (21.38 %) |  |
|  | **Q3** | 805 | 370 (45.96 %) | 323 (40.12 %) | 112 (13.91 %) |  |
|  | **Q4** | 804 | 435 (54.10 %) | 241 (29.98 %) | 128 (15.92 %) |  |
| **HOMA-IR** | **Q1** | 802 | 407 (50.75 %) | 260 (32.42 %) | 135 (16.83 %) | 2.20E-16 |
|  | **Q2** | 805 | 371 (46.09 %) | 306 (38.01 %) | 128 (15.90 %) |  |
|  | **Q3** | 803 | 291 (36.24 %) | 345 (42.96 %) | 167 (20.80 %) |  |
|  | **Q4** | 806 | 232 (28.78 %) | 353 (43.80 %) | 221 (27.42 %) |  |
| **TyG-index** | **Q1** | 794 | 380 (47.86 %) | 308 (38.79 %) | 106 (13.35 %) | 7.00E-04 |
|  | **Q2** | 814 | 346 (42.51 %) | 309 (37.96 %) | 159 (19.53 %) |  |
|  | **Q3** | 788 | 324 (41.12 %) | 311 (39.47 %) | 153 (19.42 %) |  |
|  | **Q4** | 820 | 251 (30.61 %) | 336 (40.96 %) | 233 (28.41 %) |  |
| **TyG-BMI** | **Q1** | 804 | 506 (62.94 %) | 217 (26.99 %) | 81 (10.07 %) | 2.20E-16 |
|  | **Q2** | 804 | 359 (44.65 %) | 298 (37.06 %) | 147 (18.28 %) |  |
|  | **Q3** | 804 | 272 (33.83 %) | 367 (45.65 %) | 165 (20.52 %) |  |
|  | **Q4** | 804 | 164 (20.40 %) | 382 (47.51 %) | 258 (32.09 %) |  |
| **TyG-WC** | **Q1** | 804 | 539 (67.04 %) | 204 (25.37 %) | 61 (7.59 %) | 2.20E-16 |
|  | **Q2** | 804 | 351 (43.66 %) | 312 (38.81 %) | 141 (17.54 %) |  |
|  | **Q3** | 804 | 259 (32.21 %) | 362 (45.02 %) | 183 (22.76 %) |  |
|  | **Q4** | 804 | 152 (18.91 %) | 386 (48.01 %) | 266 (33.08 %) |  |
| **TyG-WHtR** | **Q1** | 788 | 473 (60.03 %) | 232 (29.44 %) | 83 (10.53 %) | 2.20E-16 |
|  | **Q2** | 804 | 341 (42.41 %) | 309 (38.43 %) | 154 (19.15 %) |  |
|  | **Q3** | 806 | 291 (36.10 %) | 327 (40.57 %) | 188 (23.33 %) |  |
|  | **Q4** | 818 | 196 (23.96 %) | 396 (48.41 %) | 226 (27.63 %) |  |
| **TG/HDL-C ratio** | **Q1** | 794 | 370 (46.60 %) | 298 (37.53 %) | 126 (15.87 %) | 0.01 |
|  | **Q2** | 813 | 346 (42.56 %) | 329 (40.47 %) | 138 (16.97 %) |  |
|  | **Q3** | 800 | 315 (39.38 %) | 325 (40.63 %) | 160 (20.00 %) |  |
|  | **Q4** | 809 | 270 (33.37 %) | 312 (38.57 %) | 227 (28.06 %) |  |

**Table 3 Clinical characteristics of training and validation set in children with NAFLD**

|  | **training** | | | | **test** | | | | **P value** |
| --- | --- | --- | --- | --- | --- | --- | --- | --- | --- |
|  | **Q1** | **Q2** | **Q3** | **Q4** | **Q1** | **Q2** | **Q3** | **Q4** |  |
| age | <8 | 8~10 | 10~12 | ≥12 | <9.40 | 9.40~10.95 | 10.95~12.43 | ≥12.43 | 0.002893 |
| **Anthropometry** |  |  |  |  |  |  |  |  |  |
| Height | <138.58 | 138.58~149.50 | 149.50~159.00 | ≥159.00 | <138.30 | 138.30~149.00 | 149.00~157.00 | ≥157.00 | 0.3568 |
| Weight | <49.50 | 49.50~62.00 | 62.00~75.13 | ≥75.13 | <49.75 | 49.75~62.75 | 62.75~72.13 | ≥72.13 | 0.5108 |
| WC (cm) | <82.00 | 82.00~89.00 | 89.00~97.00 | ≥97.00 | <81.00 | 81.00~88.00 | 88.00~96.00 | ≥96.00 | 0.2834 |
| HC (cm) | <87.00 | 87.00~94.50 | 94.50~102.00 | ≥102.00 | <86.38 | 86.38~94.00 | 94.00~99.50 | ≥99.50 | 0.09129 |
| WHtR | <0.57 | 0.57~0.60 | 0.60~0.64 | ≥0.64 | <0.56 | 0.56~0.60 | 0.60~0.63 | ≥0.63 | 0.05602 |
| WHR | <0.90 | 0.90~0.94 | 0.94~0.98 | ≥0.98 | <0.56 | 0.56~0.60 | 0.60~0.63 | ≥0.63 | < 2.20E-16 |
| **Blood pressure** |  |  |  |  |  |  |  |  |  |
| SBP (mmHg) | <111.00 | 111.00~121.00 | 121.00~130.00 | ≥130.00 | <106.00 | 106.00~116.00 | 116.00~128.00 | ≥128.00 | 0.00228 |
| DBP (mmHg) | <63.00 | 63.00~70.00 | 70.00~176.00 | ≥76.00 | <63.75 | 63.75~68.00 | 68.00~74.25 | ≥74.25 | 0.08165 |
| **Glucose metabolism** |  |  |  |  |  |  |  |  |  |
| HbA1c (%) | <5.40 | 5.40~5.70 | 5.70~6.10 | ≥6.10 | <5.60 | 5.60~5.80 | 5.80~6.10 | ≥6.10 | 0.0138 |
| Fasting insulin (uIU/ml) | <11.55 | 11.55~18.70 | 18.70~28.30 | ≥28.30 | <13.43 | 13.43~18.30 | 18.30~28.50 | ≥28.50 | 0.5179 |
| 2h insulin (uIU/ml) | <25.20 | 25.20~62.05 | 62.05~119.85 | ≥119.85 | <30.05 | 30.05~60.05 | 60.05~112.05 | ≥112.05 | 0.8155 |
| Fasting glucose (mmol/L) | <5.00 | 5.00~5.30 | 5.30~5.60 | ≥5.60 | <5.20 | 5.20~5.40 | 5.40~5.70 | ≥5.70 | 0.0005737 |
| 2h glucose (mmol/L) | <6.00 | 6.00~6.70 | 6.70~7.40 | ≥7.40 | <6.30 | 6.30~6.90 | 6.90~7.60 | ≥7.60 | 0.0009138 |
| **Lipid profile** |  |  |  |  |  |  |  |  |  |
| TG (mmol/L) | <0.89 | 0.89~1.20 | 1.20~1.64 | ≥1.64 | <0.89 | 0.89~1.23 | 1.23~1.72 | ≥1.72 | 0.6889 |
| TC (mmol/L) | <3.69 | 3.69~4.21 | 4.21~4.79 | ≥4.79 | <3.77 | 3.77~4.32 | 4.32~4.90 | ≥4.90 | 0.08706 |
| HDL-C (mmol/L) | <1.03 | 1.03~1.19 | 1.19~1.38 | ≥1.38 | <0.92 | 0.92~1.13 | 1.13~1.35 | ≥1.35 | 0.001851 |
| LDL-C (mmol/L) | <2.13 | 2.13~2.51 | 2.51~2.96 | ≥2.96 | <2.02 | 2.02~2.38 | 2.38~2.78 | ≥2.78 | 0.01024 |
| **Inflammatory marker** |  |  |  |  |  |  |  |  |  |
| AST (U/L) | <21.00 | 21.00~27.00 | 27.00~38.00 | ≥38.00 | <23.00 | 23.00~29.00 | 29.00~44.00 | ≥44.00 | 0.005546 |
| ALT (U/L) | <19.00 | 19.00~30.00 | 30.00~56.00 | ≥56.00 | <19.75 | 19.75~37.50 | 37.50~74.00 | ≥74.00 | 0.007886 |
| Uric acid (umol/L) | <323.98 | 323.98~376.60 | 376.60~447.83 | ≥447.83 | <325.60 | 325.60~382.50 | 382.50~453.00 | ≥453.00 | 0.675 |
| **Cardiometabolic index** |  |  |  |  |  |  |  |  |  |
| ISI (composite) | <1.69 | 1.69~2.88 | 2.88~5.70 | ≥5.70 | <1.79 | 1.79~2.85 | 2.85~4.74 | ≥4.74 | 0.5732 |
| TyG | <8.23 | 8.23~8.53 | 8.53~8.85 | ≥8.85 | <8.22 | 8.22~8.56 | 8.56~8.94 | ≥8.94 | 0.3546 |
| TyG-BMI | <213.85 | 213.85~238.10 | 238.10~265.82 | ≥265.82 | <214.68 | 214.68~236.16 | 236.16~259.40 | ≥259.40 | 0.7262 |
| TyG-WC | <686.31 | 686.31~762.83 | 762.83~833.92 | ≥833.92 | <694.82 | 694.82~747.00 | 747.00~823.91 | ≥823.91 | 0.4262 |
| TyG-WHtR | <4.80 | 4.80~5.16 | 5.16~5.58 | ≥5.58 | <4.79 | 4.79~5.10 | 5.10~5.48 | ≥5.48 | 0.1782 |
| TG/HDL-C ratio | <1.08 | 1.08~1.45 | 1.45~1.95 | ≥1.95 | <0.75 | 0.75~1.11 | 1.11~1.78 | ≥1.78 | 1.79E-10 |
| HOMA-IR | <2.71 | 2.71~4.38 | 4.38~6.77 | ≥6.77 | <3.23 | 3.23~4.39 | 4.39~6.73 | ≥6.73 | 0.3082 |

**Table** **4 Variables in the equation**

| **Variables** | **B** | **S.E.** | **Wald** | **df** | **Sig.** | **Exp(B) (95%CI)** |
| --- | --- | --- | --- | --- | --- | --- |
| **HC** | 0.033 | 0.007 | 25.374 | 1 | 0.000 | 1.034(1.021-1.047) |
| **WHtR** | 4.688 | 0.637 | 54.226 | 1 | 0.000 | 108.609(31.189-378.212) |
| **ALT** | 0.036 | 0.002 | 256.713 | 1 | 0.000 | 1.036(1.032-1.041) |
| **HDL-C** | -0.647 | 0.199 | 10.574 | 1 | 0.001 | 0.524(0.355-0.773) |
| **ApoA1** | 0.007 | 0.001 | 31.878 | 1 | 0.000 | 1.007(1.005-1.010) |
| **ISI (composite)** | -0.015 | 0.005 | 9.114 | 1 | 0.003 | 0.986(0.976-0.995) |
| **TyG-WC** | .002 | 0.001 | 4.434 | 1 | 0.035 | 1.002(1.000-1.003) |
| **Sex** | 0.229 | 0.098 | 5.541 | 1 | 0.019 | 1.257(1.039-1.523) |
| **Constant** | -8.356 | 0.587 | 202.475 | 1 | 0.000 | 0.000 |
